# Diced Triplets Expose Neurons to RISC

**DOI:** 10.1371/journal.pgen.1002545

**Published:** 2012-02-23

**Authors:** Dobrila D. Rudnicki, Russell L. Margolis, Christopher E. Pearson, Wlodzimierz J. Krzyzosiak

**Affiliations:** 1 Division of Neurobiology, Laboratory of Genetic Neurobiology, Department of Psychiatry, Johns Hopkins University School of Medicine, Baltimore, Maryland, United States of America; 2 Department of Neurology and Program in Cellular and Molecular Medicine, Johns Hopkins University School of Medicine, Baltimore, Maryland, United States of America; 3 Program of Genetics and Genome Biology, The Hospital for Sick Children, Toronto, Canada; 4 Department of Molecular Genetics, University of Toronto, Toronto, Canada; 5 Laboratory of Cancer Genetics, Institute of Bioorganic Chemistry, Polish Academy of Sciences, Poznan, Poland; The University of North Carolina at Chapel Hill, United States of America

**Figure 1:**
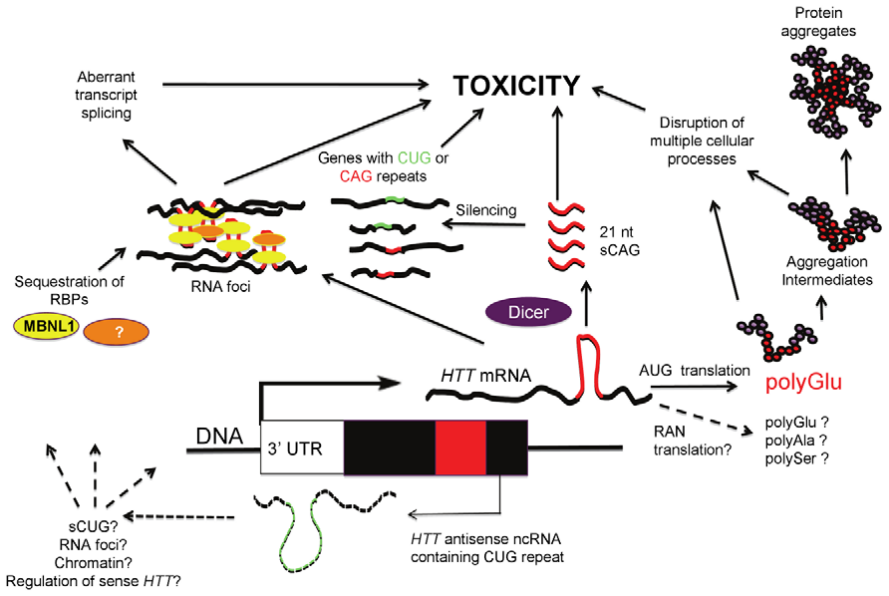
RNA contribution to HD pathogenesis. An *HTT* transcript with an expanded CAG repeat is expressed and translated into the huntingtin protein containing an expanded polyglutamine tract. The expanded polyglutamine tract leads to cell toxicity through multiple pathways. It is possible that RAN (Repeat Associated Non-ATG Translation) could generate polypeptides containing polyglutamine, polyalanine, or polyserine tracts that contribute to pathogenesis. In parallel, the expanded repeat in the transcript forms a hairpin that is cleaved by Dicer into 21-nt fragments that lead to toxicity, at least in part based on silencing of other genes that contain CUG and CAG repeats. In addition, *HTT* transcripts with expanded CAG repeats may accumulate into RNA foci, sequestering RNA binding proteins like MBNL1, leading to toxicity via mechanisms that most likely include aberrant gene splicing. A transcript antisense to the *HTT* gene may also participate in disease pathogenesis through dysregulation of the *HTT* sense transcript, formation of sCUG, and/or formation of RNA foci with protein sequestration.

Expansions of short repeats—those with units of ≤12 bp—account for as many as 40 diseases [1]–[4]. About half of these disorders arise from expanded tracts of CAG/CTG triplets, many encoding polyglutamine. Since the discovery of the first polyglutamine-encoding CAG repeat disorder in 1991 [5], the predominant hypothesis has been that pathogenesis of the CAG category is a consequence of a toxic gain-of-function of excessively long strands of polyglutamine. Polyglutamine toxicity has been most systematically explored in Huntington's disease (HD), with evidence that it influences multiple processes, including transcriptional regulation, mitochondrial energy production, and calcium regulation. This proteocentric view is undergoing considerable revision, as mounting evidence suggests toxic roles for mutant transcripts in HD [6] (Figure 1). The initial clues regarding CNG transcript toxicity emerged from studies of myotonic dystrophy type 1 (DM1). DM1 is caused by an expanded CTG repeat located in the 3′ end of the *DMPK* gene. Transcripts with long CUG repeats dysregulate the splicing factors MBNL1 and CUGBP1, leading to aberrant splicing of numerous downstream transcripts; dysfunction of these proteins directly correlates with various features of the disease phenotype. Subsequently, multiple lines of evidence have emerged that RNA toxicity contributes to the pathogenesis of other CAG/CTG disorders. Structurally, RNA with sufficiently long stretches of CUG or CAG triplets can form hairpin structures likely to influence the affinity of RNA binding proteins [7]. At least partly as a consequence of these structures and changes in protein binding, transcripts with either type of repeat may aggregate into discrete foci that include MBNL1 [8], [9]. In HD, RNA foci and misregulation of splicing have been detected in peripheral HD tissue [9]. The potential toxicity of transcripts containing long CAG tracts has been demonstrated in fly, worm, and mouse systems [10]–[14]. HDL2, a disorder clinically and pathologically similar to HD, involves CUG transcript toxicity mediated by dysregulation of MBNL1 [15], [16]. A second potential mechanism of CAG/CTG toxicity emerged from evidence that bidirectional transcription through the *HTT* repeat region [17] is a source of Dicer-generated CAG/CUG repeat siRNAs capable of targeting cellular transcripts containing complementary repeats [18].

In this issue of *PLoS Genetic*s, Bañez-Coronel and colleagues [19] provide further evidence for the involvement of *HTT* RNA and the RNAi pathway in HD pathogenesis. The authors demonstrate that overexpression of translatable and non-translatable *HTT* exon 1 constructs with expanded CAG repeats leads to Dicer-dependent production of short CAG repeat RNAs (sCAGs) with cytotoxic properties. Cytotoxic effects are triggered by expanded CAG repeats (which can form RNA hairpins), but not by expanded CAA repeats (which, like CAG, encode glutamine, but cannot form hairpins), consistent with recent findings in a fly model [11]. sCAG species were detected in Ago-2 complexes, supporting association with RNAi pathways. Antisense inhibitors of the sCAG species reverse cytotoxicity, and sCAGs were detected in R6/2 HD transgenic mice and in postmortem human HD brain tissue. sCAGs isolated from human HD tissue and then transfected into cells induced toxicity. The pathogenically relevant targets of the sCAGs remain to be determined, but initial experiments suggest several potential transcripts, including ADORA2A and MEIS2 (both reduced in HD brain tissue) and more variably DMPK, ASTN2, and ZFR, all containing either fully or partially complementary CUG and CAG repeats. Determining a more complete list of target sequences, and the extent to which downregulation is necessary or sufficient for toxicity, remain critical issues for further exploration. Curiously, the sCAG species isolated from HD models and human HD brain that induced toxicity were not a homogenous population of RNAs, but were identified in the <100-nt fraction. While cytotoxicity was sCAG-dependent (as toxicity was blocked with anti-sCAG), the relative contribution of sCAGs compared to other miRNAs in the isolated fraction is unknown. Whether Dicer is the only ribonuclease involved in sCAG production also remains to be determined.

It is noteworthy that a DM1 antisense transcript containing the repeat in the CAG orientation is also converted to 21-nt fragments that include CAG units [20], similar to the 21-nt sCAG fragments from the HD locus reported by Bañez-Coronel et al. [19]. While the function of the DM1 CAG fragments remains unknown, it was suggested that they may play a role in the abnormal chromatinization at the DM1 locus that occurs in the presence of the expansion mutation [20], raising the possibility that a similar phenomenon may also occur at other loci, such as HD, where sCNG fragments are generated. Both the DM1 and SCA7 antisense transcripts are thought to regulate their complementary sense transcripts [20], [21]. Conversely, the findings in HD by Bañez-Coronel et al. suggest that HD sCAG fragments might regulate non-HD CUG- and CAG-containing transcript levels (Figure 1), possibly through an RNA-RNA hybrid mechanism. This may occur through processes similar to the RNA-RNA hybrids formed between the expanded *DMPK* CUG repeats and the short CAG repeats in CUGBP1 mRNA, proposed to regulate the reduced CUGBP1 mRNA levels in DM1 patient muscles [22].

What regulates the expression of the *HTT* antisense and sCAG fragments is unknown but may involve epigenetic factors. The expression of the DM1 and SCA7 antisense transcripts are regulated by CTCF binding at sites proximal to the repeat and promoter regions coincident with localized chromatin modifications [20], [21]. Interestingly, CTCF binding also regulates SCA7 CAG instability [23]. The potential role of the HD CTCF site [17], [24] in regulating the expression of *HTT* or *HTT* antisense is yet to be determined.

Two recent observations complicate the interpretation of the Bañez-Coronel et al. findings [19]. First, homopolymeric polyalanine or polyserine proteins were found to be expressed via a mechanism termed Repeat Associated Non-ATG Translation (RAN) recently described by Zu et al. [25], reviewed in [26]; this raises the possibility that toxic RAN proteins could contribute to the pathogenesis induced by the AUG-free “untranslatable” *HTT* RNA fragment (Figure 1). Secondly, since the construct used by Bañez-Coronel et al. contains the entire *HTT* exon 1, it is also possible that the recently described antisense *HTT* transcript is coexpressed with the sense strand transcript [17]. Thus, the long CUG *HTT* antisense transcript might itself contribute to cytotoxicity, and/or lead to RAN-translated products, and/or influence the process by which sCAGs are generated (Figure 1).

While Bañez-Coronel et al. [19] primarily focus on the role of RNAi pathways and the toxicity of sCAGs, it is likely that toxicity induced by *HTT* sense and antisense RNAs, as in the case of *HTT* protein, involves multiple pathways, each warranting exploration. For example, what is the pathogenic effect of the aggregation of transcripts containing long CAG or CUG repeats? Might this lead to sequestration or dysregulation of splicing factors, as in DM1? Distinct proteins bind to mRNA containing CAG and CUG repeats—do properties of the transcripts, such as repeat length and the sequence of regions flanking the repeat, modulate this binding? Does the CAG repeat tract length affect transcript stability, or the efficiency of transcription or translation? What is the relationship between CAG expression level and CAG repeat length in inducing toxicity? Does RNA-mediated toxicity provide any clues to selective neuronal vulnerability in HD? Does RAN-translation arise in HD as it does in DM1 and SCA7 patient tissues? These questions demonstrate that every step of the mRNA life cycle in CAG/CTG disease warrants exploration.

Of utmost importance, the findings of Bañez-Coronel et al. [19] and others that implicate RNA in HD pathogenesis provide new leads in the search for therapeutic targets. Targeting only the mechanisms induced by expanded polyglutamine tracts may not be sufficient to stop disease pathogenesis. A comprehensive strategy to combat HD will require attention to RNA-mediated toxicity.
